# How to combine soil and plant indicators to manage nitrogen fertilisation in vineyards?

**DOI:** 10.1016/j.heliyon.2024.e40099

**Published:** 2024-11-04

**Authors:** Sylvain Vrignon-Brenas, Bénédicte Fontez, Denis Caboulet, Gabriel Ruetsch, Olivier Demarle, Aurélie Metay, Anne Pellegrino

**Affiliations:** a LEPSE, Univ Montpellier, INRAE, Institut Agro, Montpellier, France; b MISTEA, Univ. Montpellier, INRAE, Institut Agro, Montpellier, France; c Institut de La Vigne et du Vin, Narbonne, France; d Les Vignobles Foncalieu, Arzens, France; e Frayssinet, Rouairoux, France; f ABSys, Univ Montpellier, CIRAD, INRAE, Institut Agro, Montpellier, France

**Keywords:** Grapevine, Chlorophyll meter, Yeast assimilable nitrogen, Nitrogen management, Minerals, Organic

## Abstract

**Context:**

Although grapevine nitrogen (N) needs are moderate, N fertilisation in vineyards requires carefully management because berry development, aromatic composition and hence winemaking are largely influenced by N nutrition. Our objective was to develop a method for reasoned N fertilisation between and during the seasons.

**Methods:**

Specific sensitivities of soil and plant indicators to N supply and availability were estimated and indicator robustness was quantified using contrasted environmental conditions, crop management and N fertilisation in an experimental network comprising five vineyards in southern France over four successive years. The effects of four N fertilisation treatments combining contrasted amounts, forms and timing on the indicators were tested using linear models. Multiple factorial multiple analysis was used to study the effect of environmental and management factors on indicator sensitivity to mineral N fertilisation at budburst.

**Results:**

Yield, soil mineral nitrogen at budburst, chlorophyll concentration (SPAD) at veraison, leaf nitrogen content and yeast assimilable nitrogen (YAN) at harvest, were all sensitive to N fertilisation after four years. Different effects of the N treatments were observed from year three for SPAD and from year one for YAN. Additionally, SPAD and YAN indicators distinguished contrasted strategies based on different timings of N supply or the form of N fertiliser. Lastly, the response of the SPAD and YAN indicators was only slightly influenced by pedoclimatic conditions or cultural practices, at least for the variables tested here.

**Conclusion:**

Two N indicators measured from veraison (SPAD) to harvest (YAN), can provide valuable information for tactical and long-term fertilisation decisions. Notably, early SPAD information may improve technical vineyard management of interactions between fertilisation and other practices (e.g. weed control or tillage) that interfere with plant N nutrition and enable later correction of YAN values. Combining these indicators with non-destructive imaging techniques should improve N and mineral monitoring in general.

## Abbreviations

N: Nitrogen
LNC: Leaf Nitrogen Content
NUE: Nitrogen Use Efficiency
NSC: Non-structural carbohydrates
pot: Potentially reachable yield
SPAD: Soil Plant Analysis Development
SMN: Soil Mineral Nitrogen
TNC: Total Nitrogen Content
YAN: Yeast assimilable nitrogen

## 1 Introduction

Nitrogen (N) is a critical limiting element in agricultural systems. Numerous studies of both annual and perennial species have shown that low N inputs can have a negative effect on yield development and fruit metabolism, thus altering both the quantity and quality of the yield [1,2]. Grapevine N requirements are relatively moderate as they range from 45 to 65 kg ha^−1^ for a range of northern cultivars with a mean yield of 7–10 t ha^−1^ [[3], [4], [5]]. However, suboptimal N supply in many vineyards may jeopardise berry development and aromatic composition and ultimately winemaking [6,7]. High N deficits in vineyards have been shown to result from the combination of soil and canopy management practices which result in the depletion of organic matter (e.g. tillage and pruning removal), while increasing N demand (e.g. high vigour, intercropping) [8]. In this context, adapting N demand to the source by controlling canopy growth (via summer pruning or by creating conditions of mild water deﬁcit) is not sufficient *per se* and fertilisation is required for both grapevine and soil sustainability [9]. But N fertilisation needs to be carefully managed to favour grapevine metabolism and berry composition while avoiding environmental impacts through loss of soil N due to leaching and denitriﬁcation [4,10]. This implies optimising nitrogen use efficiency (NUE), which can be separated into N uptake efficiency (allocation) and N utilisation efficiency (remobilisation) [[11], [12], [13]]. The timing and amount of N fertiliser should thus be reasoned at a perennial time step to meet plant demand by considering both the initial reserve in the plant and the stock of N in the soil [14].

Different indicators can be used to monitor the level of N deficiency experienced by a plant and to guide winegrowers’ decisions. These indicators mainly include direct analysis of N content in the soil or in plant organs (leaf, petioles, wood and must) or indirect non-destructive methods based on optical sensors, which provide indirect assessment of N status either at the leaf or canopy levels. The advantages and drawbacks of these indicators for scheduling nitrogen fertilisation in vineyards are detailed below.

Soil mineral nitrogen (SMN) is not usually considered to be a reliable way of assessing plant N status and supply, as it depends on too many parameters (soil activity, water, root development, sampling method, climate, etc.), and frequent soil sampling is required over the season to capture the dynamics of nitrogen availability [11]. However, SMN analyses in winter can provide a baseline for determining the potential N supply in a vineyard, and the risks of excessive N supply and environmental issues [10]. In general, under a Mediterranean climate, N stocks in the top 30 cm of the soil that are higher than 40 kg ha^−1^ at budburst are considered sufficient to support grapevine growth [15]. But such a threshold should also account for grapevine N demand, which depends on the production objectives, on the one hand, and on weather conditions and vineyard management which influence the soil N balance, on the other.

An alternative to soil analyses is monitoring plant N status. Although visual assessment of canopy vigour and colour allows a preliminary diagnosis of overall N availability in a vineyard [16], plant N analyses are indispensable to quantify plant N status during or after the vegetative season, and the efficiency of N uptake following the application of fertiliser. For this purpose, different organs can be sampled, including the leaf (blade or petioles) from flowering to veraison, must at harvest, or pruned wood in winter. Due to intra-annual variations in leaf N content, specific thresholds should be applied at each phenological stage to interpret leaf or petiole N contents [17]. Petiole analyses have been shown to be more sensitive to supplies of nitrogen, but in turn, also to vary more among samples than leaf blade analyses [18,19]. Thus, several authors recommend leaf blade analyses to diagnose N deficit because they are assumed to be more robust across the season and across cultivars [20,21]. The supply of N to the grapevine affects fruit N accumulation, with consequences for both the total N amount and the type of amino-acids that are important fruit quality compounds [22]. The analysis of must N content at harvest can be considered as a proxy for plant N status over the fruit development period. Both total N content and yeast assimilable nitrogen (YAN) in grapes have been shown to be highly responsive to fertilisation practices [5,23]. Although the YAN values are expected to vary among grapevine varieties [13], the risk of incomplete fermentation is assumed to be limited for YAN ranging between 140 and 200 mg l^−1^ and to be zero when YAN is greater than 200 mg l^−1^. According to van Leeuwen et al. [24], in some situations, these thresholds may be lower when the goal is the production of red wine.

Lastly, indirect methods based on optical sensors that inform about N status at the leaf level (e.g. chlorophyll meters such as SPAD or an N-tester; or a flavonol meter such as Dualex) or at the canopy level (e.g. a reflectance sensor such as Greenseeker) are particularly useful because they are non-destructive, and also cheaper, faster and easier to use than the soil and plant indicators described above [10]. Chlorophyll meters measure the intensity of the green colour of the foliage, which is closely correlated with the concentration of both chlorophyll and nitrogen in the leaves [[25], [26], [27]]. Interestingly, close relationships were observed between different tools such as SPAD and Dualex [28] and N-testers [29]. All the chlorophyll meters tested proved to be very sensitive to N deficiency. For example, SPAD measurements enabled N fertilisation treatments to be distinguished well before any change in plant functioning became apparent [30]. In that respect, care must be taken when sampling the leaf (notably leaf age and exposure) for a reliable estimation of N status using a chlorophyll meter. Specific interpretation thresholds have been proposed that differ depending on the chlorophyll meter concerned. For instance, Vrignon-Brenas et al. [30] observed that SPAD readings between 30 and 35 at veraison could be considered as non-limiting for growth of cv. Sauvignon-Blanc vines. In their study, Spring and Verdenal [31] reported an optimal range of N-tester measurements at veraison of 430–580 depending on the cv., Chasselas, Pinot Noir or Gamay. Verdenal et al. [13] recommended avoiding taking measurements late in the season due to the interactive effect on measurements taken by the chlorophyll meter of factors other than N such as drought, other nutrients (e.g., magnesium, iron) and disease symptoms on the leaves [16,26].

To conclude, several indicators can be used to monitor the N status of the vine. Using more than one indicator is another way to improve the reliability of the N diagnosis of the vine over the season [32]. For instance, YAN, which is routinely measured at harvest, is usually supplemented by observations of overall plant functioning during the course of the growing season (vigour, leaf colour and bud fruitfulness). However, in most cases, the absence of universal thresholds for any of the N indicators over the season and for all vineyard situations including specific pedo-climatic conditions, genotypes and production objectives renders deciding on the timing and the amount of N difficult [33].

The objective of the present study was to identify the most appropriate N indicators for reasoned fertilisation in vineyard conditions. The method, based on the combination of multiple soil and plant indicators, relies on: (i) screening variables to identify which are responsive to mineral N fertiliser applied in spring; (ii) testing the sensitivity of the variables (hereafter referred to as ‘indicators’) to different N management strategies, i.e. different forms of N, (mineral, organic) and/or growth stage (budburst, flowering, veraison) for fertilisation, and (iii) identifying environmental and management factors that explain variations in indicators due to local conditions in order to tailor the use of N fertilisation management indicators for vineyards. F ive field experiments were conducted across a network of cultivated plots in the southern France over four consecutive years. These experiments involved four distinct N fertilisation treatments, combining different amounts (0N, 40N), forms (mineral vs. organic N) and timing (budburst, flowering and veraison) of the supply.

## 2 Material and methods

### 2.1 Experimental sites and treatments

Two experiments (hereafter Exp. 1, Exp. 2) were conducted over a period of four years (Exp. 1 from 2017 to 2020, and Exp. 2 from 2013 to 2016) in five vineyards (43°13′N to 43°57′N and 0°24′E to 2°59′E, hereafter “A”, “B”, “C”, “D” and “E”) that produce yields up to 160 hl ha^−1^ and are representative of vineyards located in south-western France. The vineyards are characterised by contrasted soil and climatic conditions (Table 1 & Fig. S3) and were 10–20 years old at the beginning of the experiments. The density of vines ranged from 4545 to 5000 plants per hectare. All the vineyards were planted with the variety Sauvignon Blanc, except for vineyard B, which was planted with Merlot. All the vines were grafted onto SO4 rootstock. Pruning was simple. Guyot and vines were trellised using vertical shoot positioning (VSP). Plants were topped once or twice before veraison to control vegetative development. Vineyards A, B and E (located in the Aude region) were irrigated to avoid a water deficit. Irrigation was not necessary in vineyards C and D because climate demand was lower (Table 1).

**Table 1:** Characteristics of vineyard experiments (CC: cover crop), climate (Cfa: humid subtropical climate, Cfb: oceanic climate, Csa: Mediterranean climate) and soil properties (SaLo: sandy loam, Lo: loam, Cl: clay, ClLO: clay loam, SaCILo: silty clay loam).

Exp	Description of the field experiment								Description of the climate during the experiments			Soil characteristics at the beginning of experiments (0–30 cm)		
	Field	N treatments	Years of experiment	Location	Cultivar	Plant density (plant per ha)	Number of buds per vine	Inter-row management	Typology (Köppen climate classification)	Mean yearly temperature during the experiment (°C)	Cumulated rainfalls (+irrigation) per year during the experiment (mm)	Texture (USDA classification)	Total nitrogen (%)	Organic matter (%)
1	A	0N; 40 MIN BB; 40 ORG BB; 40 ORG BB + FF	2017–2020	Aude (43°18′23.4″N 2°58′46.1″E)	Sauvignon Blanc	5000	13	Spontaneous CC	Cfa and Csa	15.06	910	SaLo	0.05	0.76
	B		2017–2020	Aude (43°17′28.2″N 2°59′02.7″E)	Merlot	5000	11	Spontaneous CC	Cfa and Csa	15.07	883	Lo	0.08	1.11
	C		2017–2020	Gers (43°51′10.6″N 1°43′37.0″E)	Sauvignon Blanc	4545	17	Spontaneous CC every other row	Cfa	13.58	1068	Cl	0.11	2.23
	D		2017–2020	Tarn (43°57′06.8″N 0°25′16.8″E)	Sauvignon Blanc	4545	15	Faba bean every other row	Cfb	13.37	966	CILo	0.08	1.55
2	E	0N 40 MIN BB 40 MIN VER 40 MIN FLO	2013–2016	Aude (43°12′45.4″N 2°13′00.9″E)	Sauvignon Blanc	4000	21	Spontaneous CC	Cfa and Csa	14.81	523	SaCILo	NA	0.95

For each experiment, different fertilisation strategies were tested with three randomised replicated sub-plots containing from 30 to 48 vines. In Exp. 1 (vineyards A, B, C and D), four fertilisation strategies were tested including (i) an unfertilized control (0N), (ii, iii) two applications of fertiliser of 40 U (40 KgN ha-1) close to budburst in mineral (40 MIN BB) or organic form (40 ORG BB) and (iv) the same 40 U organic fertiliser applied at budburst supplemented by foliar applications of 40 l ha^−1^ of organic N (3.6 kg N ha-1) between flowering and veraison (40 ORG BB + FF). Under the 40 N MIN BB treatment, ammonium nitrate was applied 7–20 days after budburst, while under the 40 ORG BB treatment, organic fertiliser (EO 4/3/5 + 3 GR, Frayssinet) was supplied 7–20 days before budburst. The 40 ORG BB + FF treatment consisted of the same application of organic fertiliser 7–20 days before budburst, plus four foliar applications of organic N (NUTRIBIO N 9.0.0, Frayssinet) from 10 days before flowering up to 15 days before veraison.

In Exp. 2 (vineyard E), a control treatment (0N) with no fertilisation was compared to three mineral fertilisation treatments comprising 40 kg N ha^−1^ (ammonium nitrate) supplied at budburst (40 MIN BB), or at flowering (40 MIN FLO) or at veraison (40 MIN VER).

### 2.2 Data collection

#### 2.2.1 Weather monitoring

Daily weather conditions (temperature, rainfall, global radiation, wind speed) were recorded by local weather stations over the four years of the experiment (Exp.1 and Exp.2). Potential evapotranspiration (ETP) was calculated each day according to the formula proposed by Penman [34] (eq. (1)).

(eq.1) $$E\text{TP}=\frac{m\;\text{Rn}+\rho_{a\;}c_{p\;}\left(\delta e\right)g_{a}}{\lambda_{v\;(m+}\gamma )}$$

where: m = Slope of the saturation vapor pressure curve (Pa K−1), Rn = Net irradiance (W m−2), ρa = density of air (kg m−3), cp = heat capacity of air (J kg−1 K−1), δe = vapor pressure deficit (Pa), ga = momentum surface aerodynamic conductance (m s−1), λv = latent heat of vaporisation (J kg−1), γ = psychrometric constant (Pa K−1).

#### 2.2.2 Plant measurements

##### 2.2.2.1 Yield and yeast assimilable nitrogen content at harvest

The number of bunches was counted at flowering (BBCH 65) on six plants (Exp. 2) or at harvest (BBCH 89) on 12 plants (Exp. 1) per sub-plot. At harvest (BBCH 89), the yield per plant was determined based on the bunch fresh weight of selected plants in each experiment. In addition, in Exp. 1, the yeast assimilable nitrogen (YAN) of the must was measured at harvest using a different method from the method used in Exp. 2. In Exp. 1, the amino acids and ammonium N concentrations (mg l^−1^) were analysed using a colorimetric method with o-phthalaldehyde (OPA) and N-acetylcysteine (NAC) (340 nm) and an enzymatic method with α-ketoglutarate, NADPH, glutamate dehydrogenase (340 nm), respectively. Both were assessed using a Gallery discrete analyser (Thermo Fisher Scientific, CERGY-PONTOISE, France). YAN was calculated as the sum of amino acids and ammonium content. In Exp. 2, YAN was determined using FTIR-spectroscopy (FOSS WineScan FT120, FOSS, Hillerød, Denmark).

##### 2.2.2.2 Shoot growth and nitrogen status

In both Exp. 1 and Exp. 2, in each sub-plot, five shoots were sampled on five plants (one shoot per grapevine) to determine the leaf area and the shoot dry matter (DM) content at four stages: the 10 expanded leaves stage, (BBCH 51), flowering (BBCH 65), veraison (BBCH 81) and harvest (BBCH 89). Total leaf area was determined from an allometric relation using a sub-sample of leaves (i.e. one leaf sampled every 5 leaves on the primary and the secondary axes). The leaf area of this sub-sample was measured using an electronic planimeter (Li-3100, Li-COR, USA). The sub-sample and the rest of the leaves were then oven-dried for 3 days at 60 °C before being weighed. The total leaf area was calculated by considering that the leaf DM to leaf area ratio was steady. To calculate DM, the stem was separated into stems (shoot + leaves) and fruits (berries + stem). For this purpose, each component was oven-dried for 7 days at 60 °C before being weighed.

Leaf total N content (TNC) was analysed at harvest (BBCH 89) on samples of leaf DM. The samples collected in each sub-plot were assembled and ground to pass through a 0.1 mm mesh sieve. TNC was then assayed on homogenous 5.0–7.5 mg sub-samples using a *UNICUBE*® *micro* elemental analyser (UNICUBE, France).

In addition, in Exp. 1, the chlorophyll index was measured using a chlorophyll meter manufactured by (Dualex, Force-A, Orsay, France) at flowering, veraison and harvest (BBCH65, BBCH 81 and BBCH 89, respectively) on 40 young fully expanded leaves per treatment with similar exposure to sunlight. The measurements were taken on the tenth leaf counting from the apex of the primary axis. Similar measurements were taken in Exp. 2, but using a SPAD-502, (Konica-Minolta, Osaka, Japan). Five successive SPAD measurements were taken at flowering, veraison and harvest (BBCH 65, BBCH 81 and BBCH 89, respectively) on 40 young fully expanded leaves per treatment with similar exposure to sunlight. The 40 values per plot measured in Exp.1 and in Exp. 2 were then averaged. The Dualex indexes were converted into SPAD indexes following Casa et al. [28] (2015), using the following equation eq.(2):

(eq.2) $$\text{SPAD}=-0.006\;*\;{\text{Dualex}}^{2}+1.435\;*\;\text{Dualex}-1.963$$

##### 2.2.2.3 Trunk carbohydrates and nitrogen content

Trunk non-structural carbohydrates (NSC) and total nitrogen content (TNC) were assayed in two sub-samples of five wood cores per treatment and in all the sub-plots at budburst (BBCH 0), at flowering (BBCH 65) and at harvest (BBCH 89) in year 4 in all the experiments.

Frozen trunk tissue was lyophilised for 24 h at −110 °C (Heto PowerDry LL1500, Thermo). Each tissue sample was then ground to pass through a 0.1 mm mesh sieve for sugar assays. Starch and other insoluble compounds were separated from soluble compounds in an aliquot of the powder added to a water-ethanol solution (20/80 %). In the insoluble fraction, starch was hydrolysed into glucose by autoclaving for 90 min at 110 °C, and using amyloglucosidase for 90 min at 56 °C. In the soluble fraction, a mixture of β-fructosidase, hexokinase, and phosphoglucoisomerase (GPI) was used to extract the soluble sugars. The soluble sugars (glucose, fructose and saccharose) were quantified by spectrophotometry at 340 nm according to the method proposed by Gomez et al.*,* [35] and Rolland [36]. Another aliquot of the lyophilised trunk powder was used for TNC assays using the Kjeldahl method.

##### 2.2.2.4 Soil mineral nitrogen content

Soil mineral nitrogen content (SMN) in the top 0–30 cm soil layer was determined in year 4 of the experiment, at the five unfolded leaves stage (BBCH 53). In each treatment, three replicates were sampled in each sub-plot and pooled to obtain a composite sample. Sub-samples of fresh soil were used to extract SMN in 1 M KCl solution. Nitrate and ammonium contents were determined using the Griess and Berthelot methods, respectively. A spectrophotometer (Gallery, Thermo Fisher Scientific) was used at 550 and 630 nm for the measurement [37]. Soil moisture content was determined on other soil sub-samples collected at the same soil depth after oven drying at 105 °C for 48 h.

### 2.3 Data analysis

#### 2.3.1 Global data analysis strategy

All statistical analyses summarised in Table 2 and detailed hereafter were conducted in R (Version 4.2.2) [38].

**Table 2:** Summary of the variables measured and associated statistical analyses performed depending on the experiments.

Questions	Location				Indicators and date of measurement											Topics (all variables are presented in Table S2)			treatments	statistical approach
	Exp1				Exp2	near budburst		flowering	veraison	harvest										
	A	B	C	D	E	SMN	TNC	SPAD	SPAD	SPAD	LNC	stem DM	LA	YAN	Yield	pot	itk	cli		
1a) effect of N fertilisation after 4 years of treatment	X	X	X	X	X	X	X	X	X	X	X	X	X	X	X				0N, 40 MIN BB	Mixed one-way ANOVAs (Table 2)
1b) effect of N fertilisation from the first to the fourth year of treatment	X	X	X	X	X	NA	NA	X	X		NA	NA	NA	X	NA				0N, 40 MIN BB	Mixed three-ways ANOVAs and power test (Table 3)
2a) effect of the date of fertilisation after 4 years of treatment					X	X		X	X		X	X	X	X	X				0N, 40 MIN BB, 40 MIN FLO, 40 MIN VER	One-way ANOVAs (Fig. 2, right)
2b) effect of the form of nitrogen supplied after 4 years of treatment	X	X	X	X		X		X	X		X	X	X	X	X				0N, 40 MIN BB, 40 ORG, 40 ORG + FF	Mixed one-way ANOVAs (Fig. 2, left)
3) sensitivity of selected indicators to local conditions	X	X	X	X	X	X					X			X	X	X	X	X		FMA based on topics (Figs. 3 and 4)

First, we assessed the sensitivity of a panel of potentially relevant indicators to mineral nitrogen fertilisation at budburst (Table 2, Q1a, b) after four years of N treatment (Exp. 1 & Exp. 2). For two of the indicators, chlorophyll content (SPAD), and yeast assimilable nitrogen, (YAN), we also checked for interactions between the N treatment and the year of the experiment using mixed models (detailed in section 2.3.2). In addition, a power analysis was conducted to test the power of the interaction term in the model. These two indicators were selected because (1) they provide a fast, cheap and repeatable assessment of N content (SPAD) and are routinely used by winegrowers (YAN) [13,23,39]; (2) they responded significantly to the N supply in Exp. 1 and Exp. 2.

Next, we evaluated how the N management strategies (Table 2, Q2 a, b) influenced the response of all the selected N indicators (detailed in section 2.3.3). The percentage of variation of the indicators was preferred over their absolute values in order to limit the vineyard effect. This percentage was calculated for each indicator and each vineyard experiment as the variation between the mineral fertiliser treatment at budburst (40 MIN BB) and the median value of the three sub-plots used for the control treatment with no fertiliser (0N) (see eq. (4)). The effect of the form of the N fertiliser (Exp. 1) and the timing of fertilisation (Exp. 2), were tested using a mixed model and a one-way ANOVA, in Exp. 1 and Exp. 2, respectively.

Finally, we evaluated variations in the N indicators due to the pedoclimatic conditions after four years of N treatment (Exp. 1 & Exp. 2) (Table 2, Q3). For this purpose, we used a large number of putative variables that could interfere with the response of the indicator to N fertilisation. These variables were divided into three sub-groups (hereafter termed ‘topics’), representing potentially achievable yield, climatic conditions and vineyard management (see below). A multiple factorial analysis (MFA) was performed to identify and characterise the main factors of variability of the agrosystems. A fourth topic built from the ratio described above and termed ‘indicator's response topic’ was added as a supplementary topic to discover which indicators were linked with the main factors of the agrosystems (see section 2.3.4); Table 2, Q3).

#### 2.3.2 Effects of mineral nitrogen fertilisation at budburst on various indicators (Table 2, Q1a,b)

The effect of mineral nitrogen fertilisation at budburst was studied using a mixed model. The vineyard effect, potentially caused by contrasted soil textures and climatic conditions (Table 1), was set as an uncontrolled (random) effect (in vineyards A, B, C, D and E). In contrast, the fertilisation treatment (0N vs. 40N BB) and the duration effect were considered as controlled effects and were set as fixed factors in the following model (eq. (3)):

(eq.3) Yijkl=μ+Ni+Aj+N:Aij+Pl+Eijkl

where *Yijkl* is the response variable, μ is the general mean, *Ni* and *Aj* are fixed effects (i for N treatment, j for the duration effect, respectively); P*l* ∼ N(0,σ^2^_a_) is the random effect of plot l and *Eijkl* ∼ N(0,σ^2^) the residual error where k is the repetition for plot l, treatment i and duration j.

We used the “lmerTest” package [40] to perform the analysis. Significant ANOVAs (p < 0.05), were followed by a Tukey's HSD test to assess the differences (p < 0.05) between mean values (N treatments).

Next, we tested the effect of the N treatment and of the year of the experiment on SPAD and YAN using two-way mixed ANOVAs. Due to the relatively small number of repetitions (n = 3), we also performed a power analysis (n = 100 simulations) using the package “simR” [41] to estimate the risk of accepting no interaction effect (H0) when there is an effect (this risk is equal to 1-power). When we expected an interaction and low power (<15 %), we tested the effect of N treatment in each year of the experiment using a linear mixed model. When the power was considered high enough (>15 %), we simplified the model by removing the interaction term.

#### 2.3.3 Effect of contrasted fertilisation management strategies (form of N and timing) on the sensitivity of the indicators (Table 2, Q2a,b)

The sensitivity of the selected indicators to different fertilisation treatments (mineral vs. organic, Exp. 1) or timing (budburst, flowering or veraison, Exp. 2) was tested using the percentage of change (hereafter ‘ratio’) in order to avoid an experimental effect of the vineyard. The ratio was calculated as follows:

(eq.4) $$\text{ratio}=\frac{ind\;\left(N\right)-ind\;\left(0N\right)}{ind\;\left(ON\right)}$$

where *ind(N)* is the value of the indicator for a given vineyard experiment and N treatments (40 MIN BB, 40 MIN FLO, 40 MIN VER, 40 ORG BB and 40 ORG BB + FF); *ind(0N)* is the median value of the indicator for the 0N treatment for a given vineyard experiment.

Then, for both Exp. 1 and Exp. 2, the effects of the different fertilisation treatments were compared using one-way ANOVA. When an ANOVA was significant, a Tukey's test (p = 0.05) was performed to assess the differences (p < 0.05) between mean values (N treatments).

#### 2.3.4 Effects of environmental and management factors on the sensitivity of the indicators to mineral nitrogen fertilisation at budburst (Table 2, Q3)

First, the dataset was split into three topics: climate ‘cli’, potentially achievable yield ‘pot’ and management ‘itk’ (see section 2.3.3). In order to select the principal dimensions (factors) of each topic, a multiple factorial analysis (MFA) was performed to obtain a common representation of the two first principal dimensions of each topic. The MFA was conducted with the package “FactomineR” [42]. The ‘ind’ was included as a supplementary topic to see how this topic (and its variables) are linked to the other topics used to characterise the agrosystems.

The RV-coefficient [43] was used to describe the dataset and the link between topics. The RV-coefficient consists in measuring similarity between two matrices (configurations) or is a multivariate generalisation of the *squared* Pearson correlation coefficient. The RV-coefficient measures the closeness of two sets of points which may each be represented in a matrix that can be interpreted as a non-centered squared coefficient of correlation between two matrices. The Lg coefficient measures the connection between topics. The Lg coefficient of a topic with itself is an indicator of the topic's dimensionality.

## 3 Results

### 3.1 Sensitivity of different indicators to mineral N fertiliser supplied in spring at the end of the four-year experiment

The effect of N fertilisation on the indicators yield, biomass, nitrogen status and carbon and nitrogen (C, N) storage at four main stages were analysed after four years of treatment (Table 3). Five indicators of the effect of N fertilisation were significant: soil mineral nitrogen (SMN) post-budburst chlorophyll content (SPAD) indexes at flowering, veraison and harvest, yield, yeast assimilable nitrogen (YAN) and leaf nitrogen content (LNC) at harvest (p < 0.05). In contrast, dry matter (DM in the whole stem and in the fruit), leaf area, trunk nitrogen content at budburst and harvest, and trunk total carbohydrate contents (starch and soluble sugars) at harvest were not significantly affected by fertilisation (p > 0.05).

**Table 3:** Summary of the effects after 4 successive years of N treatments on soil and plant nitrogen indicators at different phenological stages in Exp. 1 and Exp. 2. The effects of treatments (0N: control with no fertilisation and 40 MIN BB: spring mineral fertilisation) were tested using one-way ANOVA, in which the vineyard effect was considered as a random effect (α = 0.05, n = 15) (*p-value* > 0.05: ‘ns’; *p-value* < 0.05: ‘∗’; *p-value* < 0.01: ‘∗∗’; *p-value* < 0.001: ‘∗∗∗’). When a significant effect was observed, the ratio (see eq. (4)) of each variable concerned was calculated. Values are the mean ± confidence interval. ‘///’: no test was conducted.

N indicators and phenological stages of measurement		N treatment		N treatment effect	ratio (%)
		0N	40 MIN BB		
Soil mineral nitrogen (kg ha-1)	post-budburst	31.60 ± 10.17	42.52 ± 9.15	∗	78 ± 85
Trunk nitrogen content (%)		0.33 ± 0.04	0.32 ± 0.04	ns	///
SPAD indexes	flowering	28.52 ± 4.15	30.28 ± 4.08	∗	7 ± 4
	veraison	32.57 ± 3.65	35.31 ± 3.69	∗∗∗	8 ± 2
	harvest	33.55 ± 4.37	36.22 ± 3.13	∗	9 ± 4
Yield (t ha-1)	harvest	15.04 ± 1.24	16.54 ± 1.17	∗	11 ± 16
Yeast assimilable nitrogen (mg l-1)		101.38 ± 38.4	128.69 ± 49.8	∗	39 ± 16
Leaf nitrogen content (%)		1.86 ± 0.19	1.99 ± 0.18	∗∗	7 ± 4
Whole-stem DM (t ha-1)		8.38 ± 1.58	8.52 ± 1.10	ns	///
Fruit DM (t ha-1)		5.49 ± 1.49	5.46 ± 1.12	ns	///
Leaf area (m^2^)		2.10 ± 0.69	1.92 ± 0.50	ns	///
Trunk carbohydrate content (mg g-1)		90.90 ± 25.96	108.00 ± 32.08	ns	///
Trunk nitrogen content (%)		0.28 ± 0.03	0.28 ± 0.03	ns	///

SMN in the top soil layer at budburst was the earliest indicator influenced by the spring mineral N fertilisation undertaken in the preceding year. SMN increased by +78 % (Table 3) on average in the 40 MIN BB treatment compared to in the control treatment with no fertiliser (0N). However, the high variability of the increase in SMN after fertilisation between the vineyards (from 0 % to 219 %, for vineyards D and A, respectively, data not shown) underlined the inconsistent response of this indicator.

SPAD indexes at veraison varied from 25 to 43 depending on the vineyard and on the treatment (data not shown). Spring fertilisation increased the SPAD indexes at the three stages (flowering, veraison, harvest) by 8 % on average. Similarly, LNC measured at harvest varied from 1.31 to 2.47 gN per 100 g of DM depending on the vineyard and on the treatment (data not shown). LNC was significantly higher (7 % on average) under the treatment including fertilisation. Grapevine yield at the end of the four-year experiment increased by about 11 % thanks to fertilisation. However, as observed for SMN, the change in yield varied with the vineyard from −12 % in vineyard B to +73 % in vineyard A, data not shown). Lastly, the YAN content of the must increased by 39 % when N fertiliser was applied.

### 3.2 Capacity of the indicators to distinguish different forms or timings of N fertilisation at the end of the four-year experiment

**Fig. 1:**
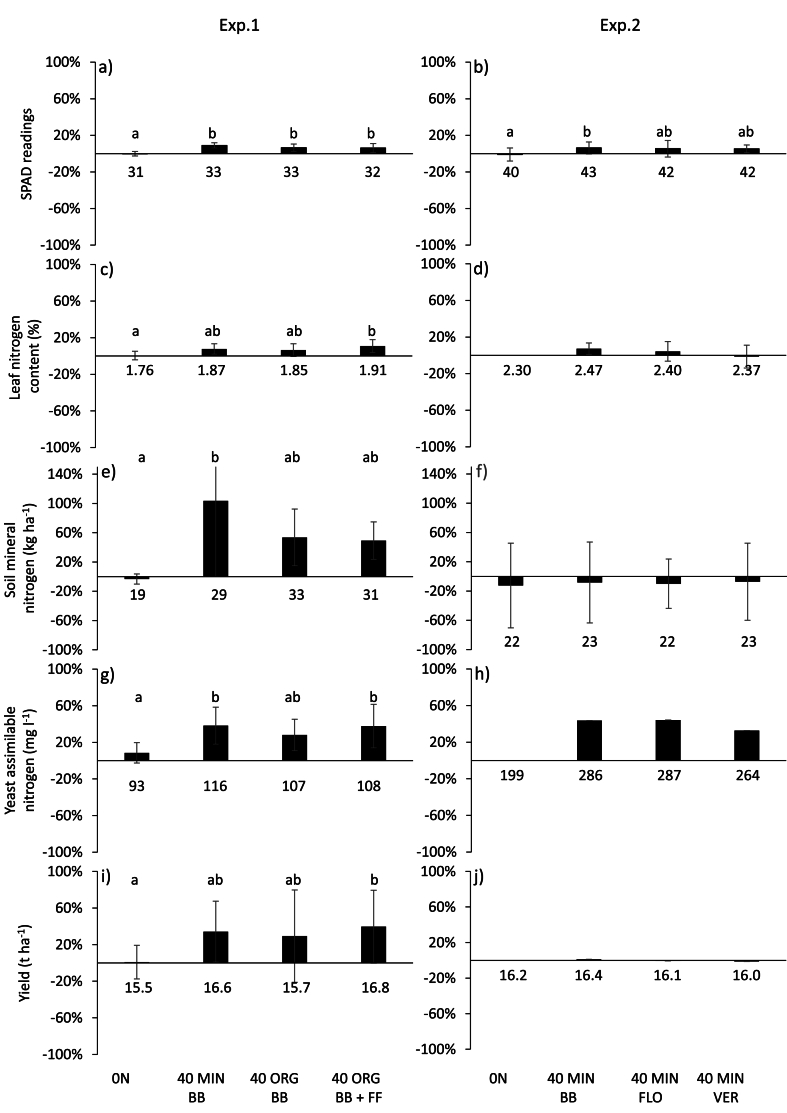
Ratio of plant and soil nitrogen indicators between the N fertilisation treatments and the control treatment with no fertiliser (eq. (4)): SPAD at veraison (a, b), leaf N content at harvest (c, d), Soil mineral nitrogen content (SMN) post-budburst (e, f), yeast assimilable nitrogen (YAN) content (g, h) and yield (i, j) at harvest. The ratios were calculated for the fourth year of N fertilisation supplied at budburst both in mineral form and in organic form (Exp.1; control with no fertilisation; 0N; mineral fertilisation in spring: 40 MIN BB; organic fertilisation in spring: 40 ORG and organic fertilisation in spring plus foliar application of organic fertiliser before veraison: 40 ORG + FF) or supplied in a mineral form with different timing (Exp. 2; control no fertilisation: 0N, mineral fertilisation in spring: 40 MIN BB; mineral fertilisation at flowering: 40 MIN FLO and mineral fertilisation at veraison: 40 MIN VER). The same letters above the bars indicate homogenous groups after Tukey's test (P < 0.05). Vertical lines indicate the confidence intervals (p = 0.05; n = 3 and n = 12 for Exp. 1 and Exp. 2, respectively). For each Exp. and indicator, the value below the histogram represents the mean value of the treatment. Due to the absence of biological repetitions, no statistical test was performed for yield and YAN content in Exp. 2 (h and j).

In the previous section, we determined which indicators were sensitive to a 40-kg mineral N fertilisation at budburst (40 MIN BB). In Exp. 1 (Fig. 1a–c, e, g, i), two other N treatments were tested, both based on organic N fertilisation at budburst (40 ORG BB), but one of them included an additional foliar application of organic fertiliser before veraison (40 ORG BB + FF). The chlorophyll content (SPAD) index showed similar increases (+9 %) for the three fertiliser treatments (40 MIN BB, 40 ORG BB and 40 ORG BB + FF) compared to the control treatment with no fertiliser (0N) (Fig. 1a). In contrast, compared to the control treatment, the leaf nitrogen content (LNC) only increased in the case of organic fertilisation including the foliar application (40 ORG BB + FF) (+8 %; Fig. 1c). The soil mineral nitrogen (SMN) content was also higher with 40 MIN BB than in the control (+104 %) but intermediate in the organic N treatments (40 ORG BB and 40 ORG BB + FF; Fig. 1e). Finally, when compared to the control, the yield for 40 ORG BB + FF was higher (+40 %) as was the yeast assimilable nitrogen (YAN) content with 40 MIN BB and 40 ORG BB + FF treatments (+38 ± 20 % and +40 ± 40 %, respectively) (Fig. 1g and i).

In Exp. 2 (Fig. 1b–d, f, h, j), we tested different timings of N mineral fertilisation (spring, flowering and veraison). A limited effect of the timing of fertilisation (40 MIN BB, 40 MIN FLO and 40 MIN VER) was observed compared to the control treatment after 4 years for the different indicators. Only the SPAD index was lower in the control treatment (-5 % compared to treatments with fertiliser), whatever the timing of fertilisation (Fig. 1b). Due to the lack of biological repetition, the potential effect of treatments could not be tested on the YAN content and yield at harvest.

### 3.3 Earliness of the indicator's responses to nitrogen supply over the four years

After four years of fertilisation, chlorophyll content (SPAD) readings and yeast assimilable nitrogen (YAN) at harvest were seen to be the two most sensitive indicators in all the experiments, regardless of the form of N supplied (mineral, organic). For this reason, we analysed the effects of interactions between the N treatment and the year of experiment on the variations undergone by these two selected indicators (Table 4).

**Table 4:** Effects of nitrogen treatment and year (after the beginning of experiment) on the SPAD index at veraison, and on YAN content at harvest. The effects of treatments and year (Y1, Y2, Y3 and Y4) were tested using two-way ANOVAs, in which the vineyard effect was considered as a random effect (α = 0.05, n = 15) (*p-value* > 0.05: ‘ns’; *p-value* < 0.05: ‘∗’; *p-value* < 0.01: ‘∗∗’; *p-value* < 0.001: ‘∗∗∗’). The same letters indicate homogenous groups after a Tukey's test (P < 0.05). Values are mean ± confident interval (α = 0.05). ‘power’ indicates the result of the *a posteriori* test of the interaction term (see section 2.3.2).

	a) SPAD indexes at veraison				b) YAN at harvest (mg l-1)			
	0N	40 MIN BB	year effect		0N	40 MIN BB	year effect	
Y1	39.97 ± 1.71	40.67 ± 1.98	c	∗∗∗	131.41 ± 34.74	157.16 ± 42.46	b	∗∗∗
Y2	34.24 ± 3.62	36.17 ± 3.23	a		129.73 ± 24.81	155.32 ± 28.07	b	
Y3	36.58 ± 1.81	38.75 ± 1.74	b		111.22 ± 43.84	126.02 ± 46.54	a	
Y4	32.57 ± 3.65	35.31 ± 3.69	a		101.43 ± 38.42	128.69 ± 49.81	a	
average (Y1 to Y4)	35.58 ± 1.56	37.53 ± 1.44			118.12 ± 16.53	141.80 ± 19.44		
treatment effect	∗∗∗		interaction: ns (power = 8 %)		∗∗		interaction: ns (power = 17 %)	

At veraison, the SPAD index (Table 4a) ranged from 33 to 41 on average, depending on the year and on the treatment. Regardless of the N treatment, the values of SPAD were lower in Y2 and Y4 than in Y1 and Y3. Interestingly, the highest SPAD values were observed in the first year of the experiment in both the control treatment (0N) and the treatments with mineral fertilisation at budburst (40 MIN BB). As expected, SPAD indexes were lower in the control treatment than in the 40N BB treatment (respectively, 35.6 and 37.5 on average for the 4 years). The interaction treatment x duration of treatment was not significant, but the estimation of power *(a posteriori*) was only 8 %. Thus, the risk of dismissing the interaction was estimated at 92 %. One way to deal with this issue was to evaluate the treatment effect for each year separately. By so doing, significant differences in the SPAD indexes (p < 0.001) were revealed between the treatments for years 3 and 4, but not for years 1 and 2 (data not shown).

At harvest, YAN content (Table 4b) ranged from 101 to 157 mg l^−1^ depending on the year and on the treatment. No significant interaction between N treatment and year was observed for YAN content, but again, the estimation of power (*a posteriori*) was only 17 %. As the estimation of the interaction effect was ten times lower than the ‘non-interaction’ effects, it was disregarded. Pairwise comparisons after ANOVA indicated that the YAN content was lower in Y3 and Y4 (<129 mg l^−1^) than in the two first years of the experiment (>130 mg l^−1^). Moreover, YAN content was significantly lower in the control treatment than in the 40N BB treatment (118 vs. 141 mg l^−1^, respectively) since the first year of N fertilisation.

### 3.4 Sensitivity of the indicators to the characteristics of the agrosystems

#### 3.4.1 Topics

To evaluate variations in the N indicators due to the pedoclimatic conditions after four years of N fertilisation, a multiple factorial analysis (MFA) was performed to identify and characterise the main factors involved in the variation in the N indicators in the different agrosystems.

**Fig. 2:**
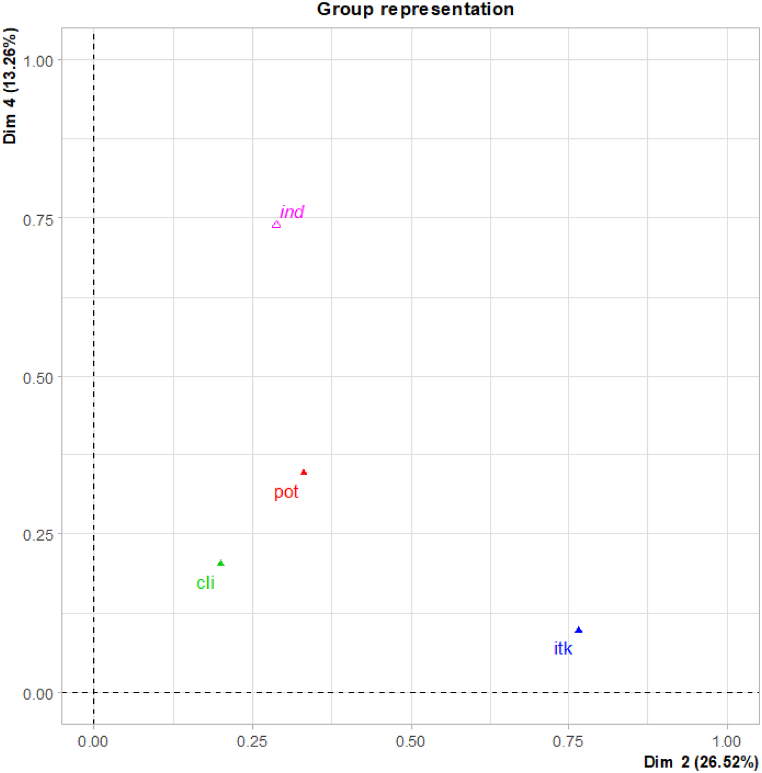
Lg (see section 2.3.4) representation of the link between topics and dimensions (2,4) of the MFA. The Lg coefficient measures the connection between topics. The Lg coefficient of a topic with itself is an indicator of the topic's dimensionality.

According to the dimensions of the inertia (Table S1), only the four first dimensions provided information on the agrosystem. After we detailed computation of the Lg coefficient (Table S1) by considering it between the indicators (the ‘ind’ topic) and each dimension of the MFA, the 'ind' topic was mainly associated with dimensions 4 and 2. We selected the plan (2,4) because, although it represented less agrosystem variability (39.8 %) compared to axis 1 (40.68 %), the ‘ind’ topic was better represented. Fig. 2 is a representation of the link between topics and plan (2,4) using Lg as coordinates. The second axis is due to the vineyard management (‘itk’ topic) and the fourth axis to the ‘ind’ topic. The two other topics are closer to each other but with a lower group representation, meaning that part of their information is not represented in this plan. Lastly, if we consider the contribution of topics, dimension 4 is driven by the vineyard potential (‘pot’ topic) and 'ind', while dimension 2 is driven by ‘itk’. The climate (‘cli’ topic) contributes less to both dimensions. Finally, we expect to observe an association only between ‘ind’, ‘pot' and ‘itk’ on the dimensions of this plan (2,4).

#### 3.4.2 Individual and quantitative and qualitative variables

**Fig. 3:**
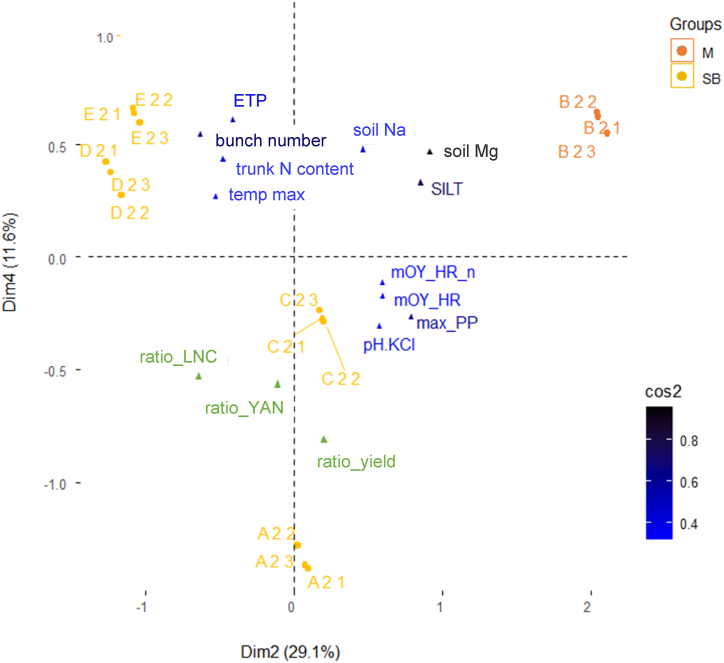
Graph of individuals, qualitative variables (cultivar: M = Merlot, SB=Sauvignon-Blanc, orange and yellow, respectively) and quantitative variables (cos^2^ > 0.3 in shades of blue) projected on dimensions 2 and 4. Supplementary variables (i.e. indicators with cos^2^ > 0.3) are also represented in green. (For interpretation of the references to colour in this figure legend, the reader is referred to the Web version of this article.)

At the variable level, the biplot in Fig. 3 identified important climate (‘cli’) topic quantitative variables between fertilisation and flowering in the plan formed by axes 2 and 4: maximum daily rainfall (max_PP) and cumulative evapotranspiration (ETP). The variables that contributed the most to potentially achievable yield (‘pot’) topic are: soil magnesium content, proportion of silt, and number of bunches (soil Mg, SILT and number of bunches, respectively). To prevent over interpretation, we chose to represent only quantitative variables with a quality of representation (cos^2^) > 35 %.

Due to the selection of quantitative variables with cos^2^ > 35 %, only three indicators (expressed as a ratio between the treatment considered and the median value of the control treatment, eq. (4)) were plotted in the correlation circle; more information on the other indicators is provided in Table S2. The first indicator was yield (‘ratio_yield’), which was highly correlated with dimension 4, the second indicator was the leaf nitrogen content (‘ratio_LNC’), which was correlated with both dimensions. Yeast assimilable nitrogen (‘ratio_YAN’) was mainly correlated with dimension 4, but to a lesser extent than yield (‘ratio_yield’).

Regarding the qualitative variables (management ‘itk’ topic), only the cultivar was represented in the correlation circle. The contribution to dimensions (2,4) and the quality of representation was also computed for the other variables (Table S2). Results showed that ‘itk’ was only represented on dimension 2 together with the cultivar (Merlot, cos^2^ = 0.84), the latter being the variable that contributed the most.

Finally, regarding the individuals (i.e. the vineyards), Fig. 3 shows that vineyard B was characterised by its specific cultivar (Merlot). This vineyard was also characterised by higher values of potential variables (soil Mg, SILT). This agrosystem was associated with low yield, and low LNC and YAN ratios. Vineyard A was mainly correlated with dimension 4 and differed from the other vineyards, with no clear variables linked to it except the small number of bunches. Vineyards D and E had a low representation and were linked to climatic conditions with high evapotranspiration, and to potential yield with a large number of bunches and high trunk N content. Vineyard C was poorly represented and was not well-characterised by the variables measured in the present study.

## 4 Discussion

### 4.1 Selection of soil and plant indicators sensitive to N supply

The N supplies in these experiments (40 kgN ha^−1^ year^−1^) are close to the standard recommendation for the Mediterranean region (20–60 KgN ha^−1^; [15]) as well as close to recommendations for other vineyard areas [5,31,44].

As reported in other studies [10,13,23,45], our results demonstrated that direct N measurements based on the top soil layer (soil mineral nitrogen, SMN) after budburst, or on the leaf (leaf nitrogen content, LNC) and must (yeast assimilable nitrogen, YAN) at harvest, made it possible to distinguish between N treatments. Non-destructive measurements such as chlorophyll content (SPAD) readings from flowering to harvest, and indirect indicators of N status such as yield were also sensitive to N treatments. No significant effect of N fertilisation was observed on the final leaf area and dry matter production, although in young potted plants, leaf area has been suggested to be an early indicator that is strongly affected by N fertilisation [14,30]. Summer pruning may have buffered the effects of fertilisation on vegetative growth. As a consequence, leaf area and shoot biomass may not have been as responsive at harvest as observed in pot experiments using plants with low N reserves.

Finally, the five indicators (SMN after budburst, SPAD at flowering, veraison and harvest, YAN and LNC at harvest and yield were all sensitive to nitrogen mineral fertilisation (40 kgN ha^−1^) applied near budburst. Consequently, these indicators may be suitable for evaluating N status in commercial vineyards. Their specific sensitivity varied from +7 % for LNC to +78 % for SMN. The SPAD readings and YAN sensitivities were intermediate, respectively +8 % and +39 %, (Table 3). While the SMN was shown to be an early indicator of nitrogen fertilisation, its response differed considerably among the vineyard experiments and between repetitions in the same vineyard. Indeed, the variation in the ratio among the vineyards/repetitions was 10 % higher than the expected variation between the N treatments (Table 3). These contrasted responses cannot be fully explained by the factors we tested (potential yield, weather conditions and vineyard management) but could be partly explained by the overall high level of nitrogen in the vineyards include in the present study. Similarly, the variations in the yield ratio between the vineyards were +5 % higher than the variations expected between the N treatments, meaning this indicator is unusable. This result is not surprising since yield depends on a multitude of environmental factors and management practices that may override the effect of fertilisation [46,47]. In the end, only three indicators among the five tested, including LNC, SPAD readings and YAN, were shown to be relevant, as their sensitivity to N fertilisation was on average 2–3 fold higher than the variations in their ratio among the vineyards. The LNC indicator was the least sensitive of the three indicators, and was also more complex and more expensive than SPAD measurements. Consequently, SPAD readings were shown to be a crucial indicator for tactical fertilisation management because they are not only cheap and easy to measure but also highly sensitive to N fertilisation. In the present study, SPAD readings were made at flowering, veraison and harvest, but SPAD was most reliable at veraison because the ratio was less variable than at flowering and harvest (Table 3). Although veraison can be considered as a late stage for fertilisation, the identification of N deficiency at this stage can still be corrected through foliar N supply to reach YAN thresholds suitable for complete fermentation. Earlier SPAD measurements at flowering, or even before, i.e. starting at the 10 expanded leaves stage, despite being more variable in the present study, would be the most suitable for medium-term corrections of the soil N status. The YAN at harvest also appeared to be a relevant indicator because it showed high sensitivity to N supply and is commonly measured by winegrowers. However, unlike SPAD readings, this indicator cannot be used for tactical fertilisation in the current year, but rather as a tool to adapt an N-fertilisation strategy, based on organic or mineral N supply, in the following year.

### 4.2 Chlorophyll content and yeast assimilable nitrogen are relevant indicators to distinguish N status in contrasted agrosystems

Our vineyards were characterised by contrasted pedoclimatic conditions (see Table 1) and by moderate or high yields, ranging from 53 to 160 hl ha^−1^. The indicators were thus measured in a wide range of situations compared to reports in the literature, but which generally correspond to high water and N supplies. Indeed, the leaf nitrogen content (LNC) ranged from 1.41 to 2.38 g per 100 g of dry matter and the chlorophyll content (SPAD) from 27 to 42 [31]. However, yeast assimilable nitrogen (YAN) values were below 140 mg l-1 in vineyards ‘A’ and ‘B’, while in vineyards ‘D’ and ‘E’, YAN reached 200 mg l-1. Yet, the low YAN values in most vineyards increase the risk of incomplete fermentation [13]. In conclusion, despite the overall high yield and N status (except for YAN) observed in our vineyards, the indicators were able to detect an additional supply of 40 KgN ha^−1^. These results suggest that indicators such as SPAD readings and YAN can cover a wide range of N conditions and can even distinguish between N treatments in the case of high levels of N.

Although fertilisation with 40 KgN ha-1 led to a significant increase in the indicators in both Exp. 1 and Exp. 2, the response rates varied with the N fertilisation strategy (organic or mineral fertiliser, timing of N application) (Fig. 1). Yet, under our high initial N conditions - as observed in the control treatment (0N) - mineral fertilisation at budburst (40 MIN BB) appeared to be more efficient than later mineral fertilisation, i.e. after flowering, and also more efficient than organic fertilisation at budburst, except when organic fertilisation was supplemented with N foliar application after flowering. In addition, the study of year × treatment interaction (data not shown) showed that mineral N fertilisation at budburst significantly affected SPAD indexes only after two years of N supply, probably because of the high initial N status of the soil and of the plants in our study. It can also be hypothesised that the limited effect and/or delayed response to nitrogen fertilisation, contingent upon the timing or the form of N applied, stem from the internal transport of nitrogen within the plant and the mobilisation of nitrogen reserves from perennial organs [13]. Lastly, although significant, the tiny differences between no fertiliser and 40 MIN BB which reached *ca.* 3 SPAD readings, underline the need to correctly estimate mean values at the vineyard level with a sufficient number of replicates. Regarding the YAN, our results showed that this indicator was sensitive to N fertilisation from the first year of treatment on. Due to the absence of replicates, no statistical analysis was performed to compare the effect of the timing of N supply (Exp.2) but YAN increased in the same range (by around 40 %) as in Exp. 1. This result suggests that YAN should be used as an indicator of N supply after only a full year of N fertilisation.

Considering our dataset, we decided to use a descriptive approach of the variations in the indicators, because many factors varied simultaneously and the relatively small number of vineyard experiments and climatic years meant we were unable to correctly estimate the effect of each individual factor. For this reason, a multiple factorial analysis (MFA) was used to describe the dataset and determine which variables were the most structuring plus how the indicators were positioned within this structure (Fig. 3). With the exception of yield at the bottom of dimension 4 (and to a lesser extent, the YAN), N indicators, particularly SPAD, were poorly represented (cos^2^ < 0.4) in this MFA (Table S2). Although the soil mineral nitrogen (SMN) was also poorly represented in this MFA, the high variability of this indicator means it is not easy to use and hence not relevant. In our conditions, the yield increased on average by 1.5 t ha^−1^ with the 40 MIN BB treatment (Table 3), leading to a nitrogen use efficiency (NUE) of 0.41 t per kg of N applied, similar to values reported elsewhere [5]. This underlines the interest of considering yield as an indicator of N nutrition. However, yield was also negatively correlated with the variables potentially reachable yield ‘pot’ and climate ‘cli’ topics and notably included cumulated potential evapotranspiration “ETP”, “number of bunches” and “proportion of silt in the soil” (Table S2). The impact of pedoclimatic conditions and overall plant functioning on yield is well known [48,49]. Nevertheless, the negative effect of high climatic demand, which can favour water deficit, on yield response to N, is not surprising. Lastly, as the number of bunches is the main yield component, high inflorescence differentiation under favourable climatic conditions, is likely to have buffered the impact of N treatment in the present study, as the N status was rather high regardless of the N treatments. However, other correlations between yield and variables such as soil magnesium and silt contents are more difficult to interpret. To conclude, the yield ratio response to N should be carefully assessed because it can vary due to several climate/plant/and soil factors. In contrast, SPAD was shown to be a relatively stable indicator, as its responses to N were independent of the agrosystem variables tested in our experimental conditions grouped in the ‘pot’ and ‘cli’ topics.

### 4.3 Towards a strategy to manage N fertilisation over seasons and years

Our study clearly underlines the interest of combining different N indicators for fertilisation management since they were shown to be responsive to N supply from year 2 on (Table 2, Table 3) in a variety of pedoclimatic conditions (Table 1). We also showed that, at least for the most common characteristics of an agrosystem, the response of indicators was only slightly influenced by the agrosystem variables, especially chlorophyll content (SPAD) readings (Fig. 3). The y east assimilable nitrogen (YAN), which is routinely measured by winegrowers, responded to N supply with a limited effect of the agrosystems parameters. However, as it was measured late in the cropping season (at harvest), this indicator cannot be used for tactical management but rather to predict fertiliser requirements for the following year. However, certain additional precautions need to be taken or limitations acknowledged before using these indicators to manage nitrogen fertilisation in vineyards. Firstly, a ratio based on median values necessitates the existence of at least one unfertilized area as a control, a requirement that may not align well with commercial practices. Secondly, our experiment was marked by relatively high potential yield. Although the response of indicators was significant in the case of a satisfactory supply of N, the relationship could be non-linear in the case of low N supply and a distorted interpretation could negatively affect N fertilisation. Thirdly, although the SPAD index is a non-destructive tool to monitor grapevine nitrogen status and is widely used by agricultural consultants, some limitations have nevertheless been reported. (1) The SPAD index is less sensitive at detecting early stages of nitrogen deficiency, and the readings become more variable as the deficiency progresses [50]. (2) SPAD index readings are influenced by different factors such as light intensity, leaf age, and temperature [51]. (3) SPAD index values vary among grapevine cultivars and/or among types of rootstock, and consequently, the same SPAD readings may not reflect the same nitrogen status in different cultivars. (4) The SPAD index can be influenced by other confounding factors such as water stress, disease, and pest damage, which can reduce its accuracy in predicting grapevine nitrogen status [45,52]. Using a ratio may be a good way to overcome these limitations; but despite these limitations, provided it is combined with other techniques, the SPAD index is still a useful tool for monitoring grapevine nitrogen.

Our study suggests that using a set of indicators rather than a single indicator, would help winegrowers manage N fertilisation throughout the season and at multi-annual scale. In addition to the indicators used in the present study, other non-destructive methods are also possible. The normalized difference vegetative index (NDVI), calculated from reflectance sensors, has been shown to be effective in assessing the spatial variability of canopy nitrogen status and to optimise agronomic practices in specific field conditions [10,13]. However, this index combines information such as leaf density (linked to vine vigour, that is influenced by other factors, not only nitrogen status) and the intensity of leaf colour (related to nitrogen status and, to a lesser extent, to the crop variety of vine). Some studies suggest that combining SPAD and NDVI measurements could serve as a tool for nitrogen management [53]. Recent studies using near-infrared spectroscopy (NIRS) technologies show promise for nitrogen measurement, along with other nutrients, using wavelengths from 780 nm to 2500 nm, thereby potentially mitigating the cultivar effects observed in SPAD readings [54]. Nonetheless, this technology requires extensive calibration and a larger dataset than the one we used in the present study. Once the relationship between leaf nitrogen content and wavelength has been established, hyperspectral cameras could be used to map nitrogen status [55].

## 5 Conclusion

Our study focused on the selection of five soil and plant indicators that are sensitive to nitrogen (N) supplies in vineyards. We found that direct measurements of N using soil, leaf, and must indicators were responsive to N supply, as demonstrated by previous studies. Additionally, indirect measurements such as chlorophyll content (SPAD) readings at veraison, and yield were positively correlated with N supply. Although at first glance, these indicators appear promising for evaluating N status in commercial vineyards, we have shown that some of them require careful consideration. Indeed, variations in soil mineral nitrogen (SMN) and yield ratio indicators between vineyards were higher than the expected variations between N treatments, meaning these indicators are not useable. In contrast, leaf N content, SPAD readings and yeast assimilable nitrogen (YAN), particularly the two latter, which are easier to handle, appear to be valuable indicators for tactical N fertilisation management. Indeed, despite the overall high N status in our experiments, SPAD readings and YAN indicators were still able to detect an additional 40 kgN ha-1 supply after two years, regardless of the growth stage (budburst to veraison) or of the form of N (mineral, organic) applied. These two indicators, expressed as a ratio of unfertilized control treatments, were also shown to be stable regardless of the period of application (budburst or budburst + veraison) in all the agrosystems tested, which varied in terms of climate, soil and management. Finally, these indicators, which can be measured from early (SPAD) to late (YAN) stages of the seasons, provide valuable information for tactical and long-term fertilisation decisions. Notably, early SPAD information can help analyse fertilisation in interaction with other practices such as weed control or tillage that interfere with the plant's nitrogen nutrition and correct YAN values later on. Combining these indicators with other new non-destructive techniques based on imagery may enhance nitrogen, and more widely mineral, monitoring.

## CRediT authorship contribution statement

**Sylvain Vrignon-Brenas:** Writing – original draft, Visualization, Formal analysis, Data curation, Conceptualization. **Bénédicte Fontez:** Writing – original draft, Visualization, Supervision, Project administration, Methodology, Funding acquisition, Formal analysis, Data curation, Conceptualization. **Denis Caboulet:** Supervision, Project administration, Investigation, Funding acquisition, Data curation. **Gabriel Ruetsch:** Investigation. **Olivier Demarle:** Supervision, Project administration, Funding acquisition. **Aurélie Metay:** Writing – original draft, Project administration, Methodology, Investigation, Funding acquisition, Conceptualization. **Anne Pellegrino:** Writing – original draft, Supervision, Project administration, Methodology, Investigation, Funding acquisition, Conceptualization.

## Data availability

Data are available upon reasonable request.

## Declaration of competing interest

The authors declare that the research was conducted in the absence of any commercial or financial relationships that could be construed as a potential conflict of interest.
