# Supplementary figures and images for: How to combine soil and plant indicators to manage nitrogen fertilisation in vineyards?
Source: Heliyon. 2024 Nov 4;10(21):e40099. doi: 10.1016/j.heliyon.2024.e40099 (PMC11582434; doi:10.1016/j.heliyon.2024.e40099)

## Slide 1
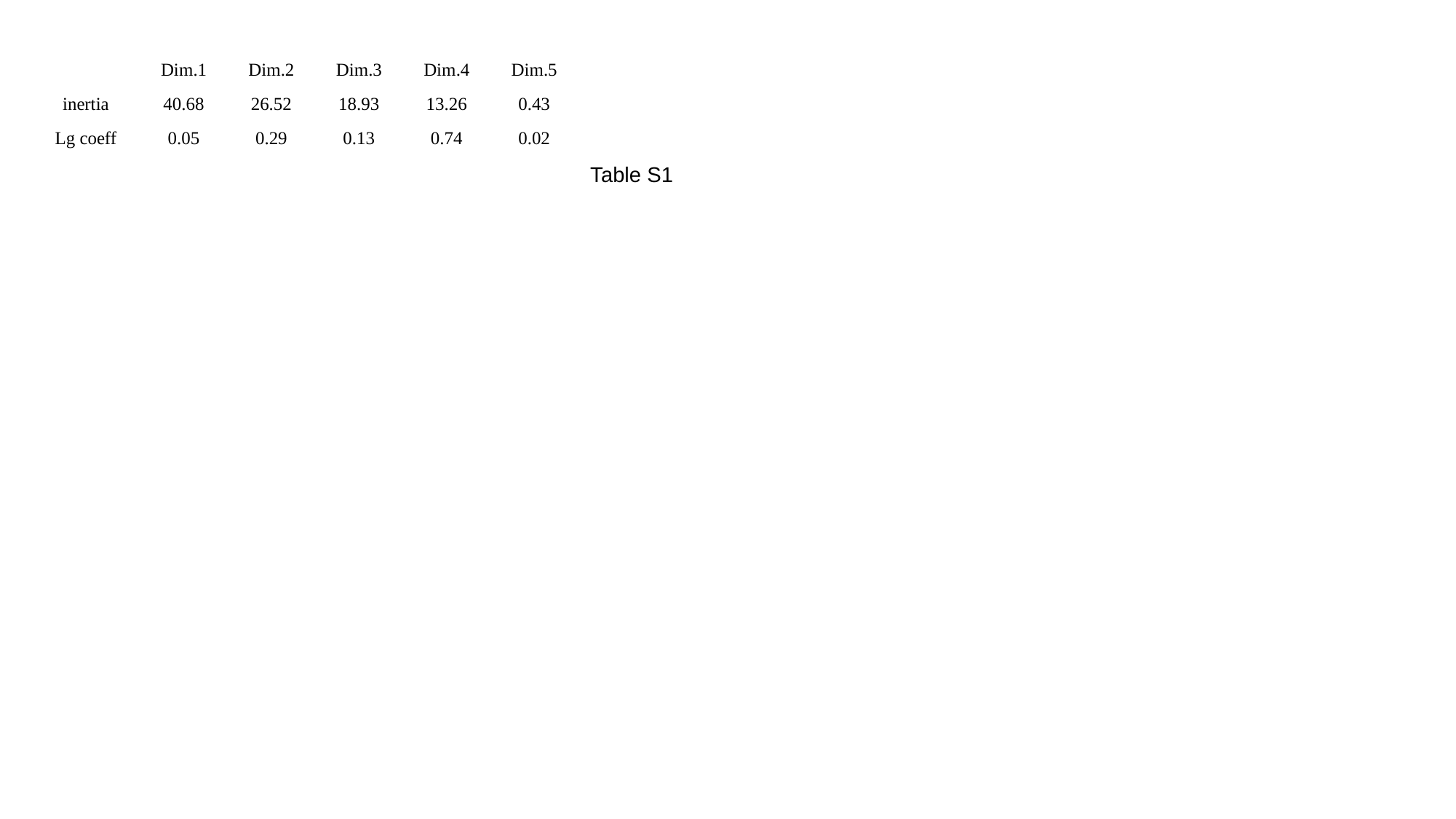

| | Dim.1 | Dim.2 | Dim.3 | Dim.4 | Dim.5 |
| --- | --- | --- | --- | --- | --- |
| inertia | 40.68 | 26.52 | 18.93 | 13.26 | 0.43 |
| Lg coeff | 0.05 | 0.29 | 0.13 | 0.74 | 0.02 |
Table S1

Supplement: Multimedia component 1 [file mmc1.pptx]
