# Supplementary material for: How to combine soil and plant indicators to manage nitrogen fertilisation in vineyards?
Source: Heliyon. 2024 Nov 4;10(21):e40099. doi: 10.1016/j.heliyon.2024.e40099 (PMC11582434; doi:10.1016/j.heliyon.2024.e40099)
Supplement: Multimedia component 2 [file mmc2.pptx]

## Slide 1
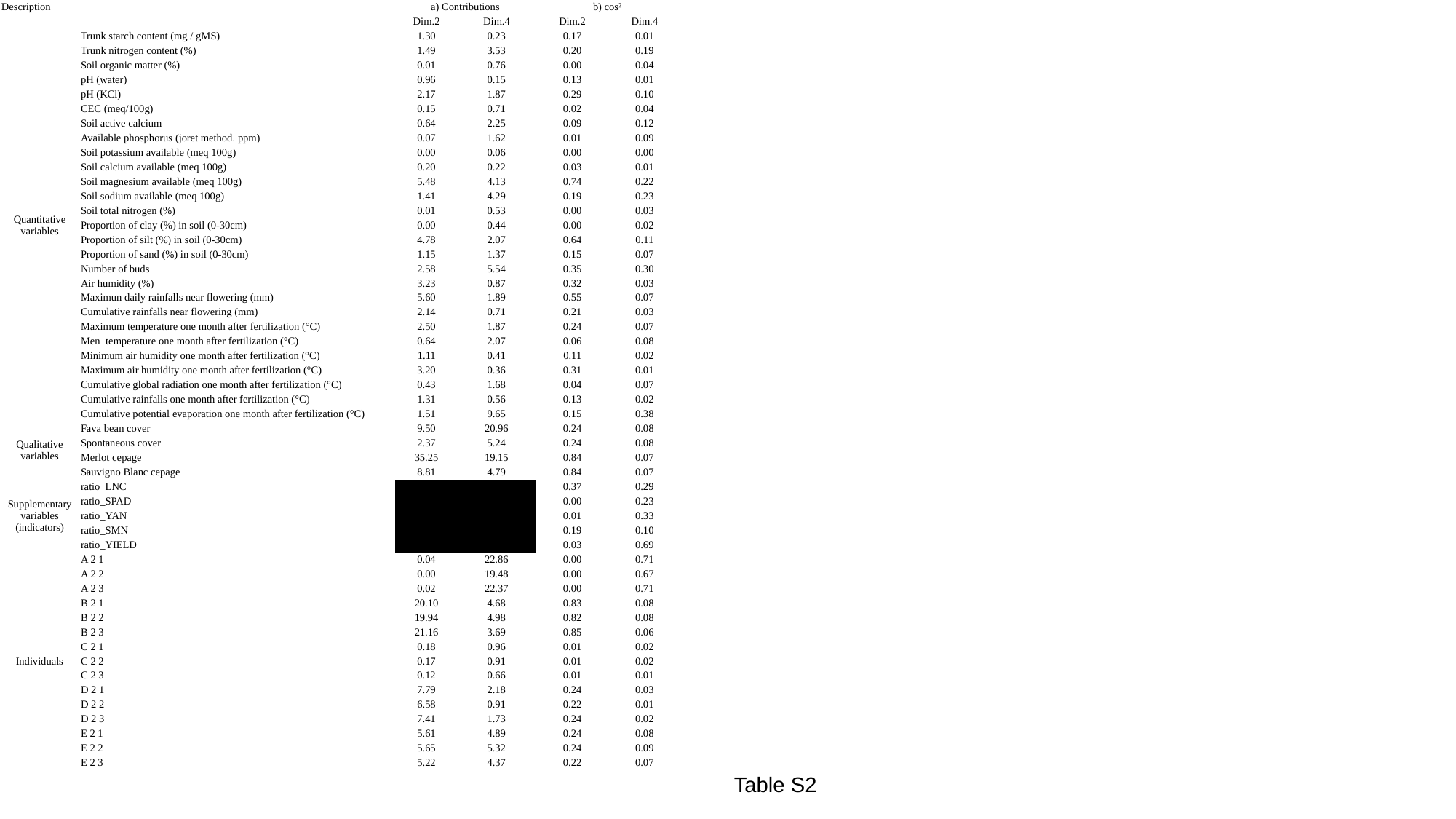

| Description | | a) Contributions | | b) cos² | |
| --- | --- | --- | --- | --- | --- |
| | | Dim.2 | Dim.4 | Dim.2 | Dim.4 |
| Quantitative variables | Trunk starch content (mg / gMS) | 1.30 | 0.23 | 0.17 | 0.01 |
| | Trunk nitrogen content (%) | 1.49 | 3.53 | 0.20 | 0.19 |
| | Soil organic matter (%) | 0.01 | 0.76 | 0.00 | 0.04 |
| | pH (water) | 0.96 | 0.15 | 0.13 | 0.01 |
| | pH (KCl) | 2.17 | 1.87 | 0.29 | 0.10 |
| | CEC (meq/100g) | 0.15 | 0.71 | 0.02 | 0.04 |
| | Soil active calcium | 0.64 | 2.25 | 0.09 | 0.12 |
| | Available phosphorus (joret method. ppm) | 0.07 | 1.62 | 0.01 | 0.09 |
| | Soil potassium available (meq 100g) | 0.00 | 0.06 | 0.00 | 0.00 |
| | Soil calcium available (meq 100g) | 0.20 | 0.22 | 0.03 | 0.01 |
| | Soil magnesium available (meq 100g) | 5.48 | 4.13 | 0.74 | 0.22 |
| | Soil sodium available (meq 100g) | 1.41 | 4.29 | 0.19 | 0.23 |
| | Soil total nitrogen (%) | 0.01 | 0.53 | 0.00 | 0.03 |
| | Proportion of clay (%) in soil (0-30cm) | 0.00 | 0.44 | 0.00 | 0.02 |
| | Proportion of silt (%) in soil (0-30cm) | 4.78 | 2.07 | 0.64 | 0.11 |
| | Proportion of sand (%) in soil (0-30cm) | 1.15 | 1.37 | 0.15 | 0.07 |
| | Number of buds | 2.58 | 5.54 | 0.35 | 0.30 |
| | Air humidity (%) | 3.23 | 0.87 | 0.32 | 0.03 |
| | Maximun daily rainfalls near flowering (mm) | 5.60 | 1.89 | 0.55 | 0.07 |
| | Cumulative rainfalls near flowering (mm) | 2.14 | 0.71 | 0.21 | 0.03 |
| | Maximum temperature one month after fertilization (°C) | 2.50 | 1.87 | 0.24 | 0.07 |
| | Men temperature one month after fertilization (°C) | 0.64 | 2.07 | 0.06 | 0.08 |
| | Minimum air humidity one month after fertilization (°C) | 1.11 | 0.41 | 0.11 | 0.02 |
| | Maximum air humidity one month after fertilization (°C) | 3.20 | 0.36 | 0.31 | 0.01 |
| | Cumulative global radiation one month after fertilization (°C) | 0.43 | 1.68 | 0.04 | 0.07 |
| | Cumulative rainfalls one month after fertilization (°C) | 1.31 | 0.56 | 0.13 | 0.02 |
| | Cumulative potential evaporation one month after fertilization (°C) | 1.51 | 9.65 | 0.15 | 0.38 |
| Qualitative variables | Fava bean cover | 9.50 | 20.96 | 0.24 | 0.08 |
| | Spontaneous cover | 2.37 | 5.24 | 0.24 | 0.08 |
| | Merlot cepage | 35.25 | 19.15 | 0.84 | 0.07 |
| | Sauvigno Blanc cepage | 8.81 | 4.79 | 0.84 | 0.07 |
| Supplementary variables (indicators) | ratio\_LNC | | | 0.37 | 0.29 |
| | ratio\_SPAD | | | 0.00 | 0.23 |
| | ratio\_YAN | | | 0.01 | 0.33 |
| | ratio\_SMN | | | 0.19 | 0.10 |
| | ratio\_YIELD | | | 0.03 | 0.69 |
| Individuals | A 2 1 | 0.04 | 22.86 | 0.00 | 0.71 |
| | A 2 2 | 0.00 | 19.48 | 0.00 | 0.67 |
| | A 2 3 | 0.02 | 22.37 | 0.00 | 0.71 |
| | B 2 1 | 20.10 | 4.68 | 0.83 | 0.08 |
| | B 2 2 | 19.94 | 4.98 | 0.82 | 0.08 |
| | B 2 3 | 21.16 | 3.69 | 0.85 | 0.06 |
| | C 2 1 | 0.18 | 0.96 | 0.01 | 0.02 |
| | C 2 2 | 0.17 | 0.91 | 0.01 | 0.02 |
| | C 2 3 | 0.12 | 0.66 | 0.01 | 0.01 |
| | D 2 1 | 7.79 | 2.18 | 0.24 | 0.03 |
| | D 2 2 | 6.58 | 0.91 | 0.22 | 0.01 |
| | D 2 3 | 7.41 | 1.73 | 0.24 | 0.02 |
| | E 2 1 | 5.61 | 4.89 | 0.24 | 0.08 |
| | E 2 2 | 5.65 | 5.32 | 0.24 | 0.09 |
| | E 2 3 | 5.22 | 4.37 | 0.22 | 0.07 |
Table S2
