# Supplementary material for: How to combine soil and plant indicators to manage nitrogen fertilisation in vineyards?
Source: Heliyon. 2024 Nov 4;10(21):e40099. doi: 10.1016/j.heliyon.2024.e40099 (PMC11582434; doi:10.1016/j.heliyon.2024.e40099)
Supplement: Multimedia component 3 [file mmc3.pptx]

## Slide 1
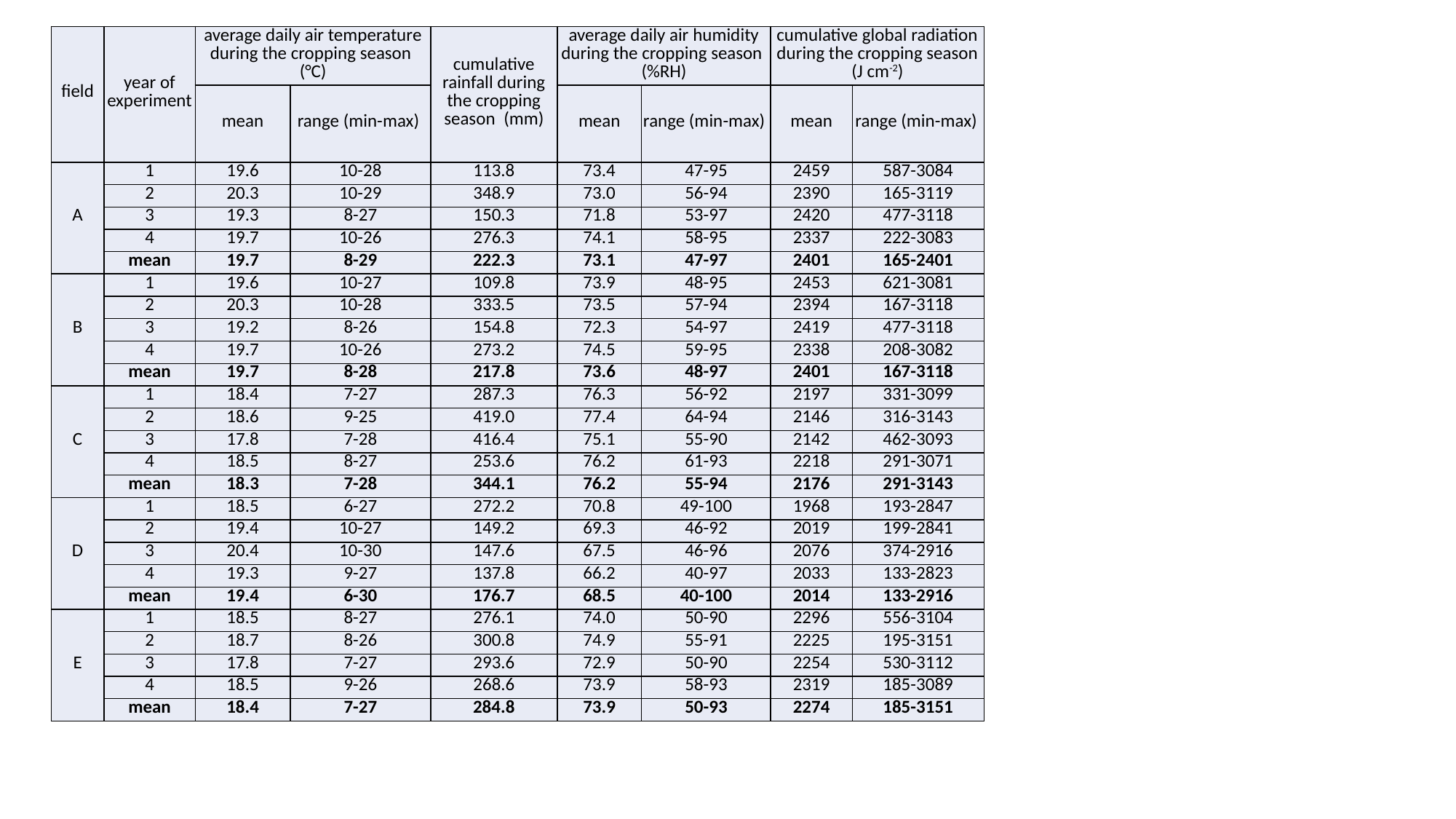

| field | year of experiment | average daily air temperature during the cropping season (°C) | | cumulative rainfall during the cropping season  (mm) | average daily air humidity during the cropping season (%RH) | | cumulative global radiation during the cropping season (J cm-2) | |
| --- | --- | --- | --- | --- | --- | --- | --- | --- |
| | | mean | range (min-max) | | mean | range (min-max) | mean | range (min-max) |
| A | 1 | 19.6 | 10-28 | 113.8 | 73.4 | 47-95 | 2459 | 587-3084 |
| | 2 | 20.3 | 10-29 | 348.9 | 73.0 | 56-94 | 2390 | 165-3119 |
| | 3 | 19.3 | 8-27 | 150.3 | 71.8 | 53-97 | 2420 | 477-3118 |
| | 4 | 19.7 | 10-26 | 276.3 | 74.1 | 58-95 | 2337 | 222-3083 |
| | mean | 19.7 | 8-29 | 222.3 | 73.1 | 47-97 | 2401 | 165-2401 |
| B | 1 | 19.6 | 10-27 | 109.8 | 73.9 | 48-95 | 2453 | 621-3081 |
| | 2 | 20.3 | 10-28 | 333.5 | 73.5 | 57-94 | 2394 | 167-3118 |
| | 3 | 19.2 | 8-26 | 154.8 | 72.3 | 54-97 | 2419 | 477-3118 |
| | 4 | 19.7 | 10-26 | 273.2 | 74.5 | 59-95 | 2338 | 208-3082 |
| | mean | 19.7 | 8-28 | 217.8 | 73.6 | 48-97 | 2401 | 167-3118 |
| C | 1 | 18.4 | 7-27 | 287.3 | 76.3 | 56-92 | 2197 | 331-3099 |
| | 2 | 18.6 | 9-25 | 419.0 | 77.4 | 64-94 | 2146 | 316-3143 |
| | 3 | 17.8 | 7-28 | 416.4 | 75.1 | 55-90 | 2142 | 462-3093 |
| | 4 | 18.5 | 8-27 | 253.6 | 76.2 | 61-93 | 2218 | 291-3071 |
| | mean | 18.3 | 7-28 | 344.1 | 76.2 | 55-94 | 2176 | 291-3143 |
| D | 1 | 18.5 | 6-27 | 272.2 | 70.8 | 49-100 | 1968 | 193-2847 |
| | 2 | 19.4 | 10-27 | 149.2 | 69.3 | 46-92 | 2019 | 199-2841 |
| | 3 | 20.4 | 10-30 | 147.6 | 67.5 | 46-96 | 2076 | 374-2916 |
| | 4 | 19.3 | 9-27 | 137.8 | 66.2 | 40-97 | 2033 | 133-2823 |
| | mean | 19.4 | 6-30 | 176.7 | 68.5 | 40-100 | 2014 | 133-2916 |
| E | 1 | 18.5 | 8-27 | 276.1 | 74.0 | 50-90 | 2296 | 556-3104 |
| | 2 | 18.7 | 8-26 | 300.8 | 74.9 | 55-91 | 2225 | 195-3151 |
| | 3 | 17.8 | 7-27 | 293.6 | 72.9 | 50-90 | 2254 | 530-3112 |
| | 4 | 18.5 | 9-26 | 268.6 | 73.9 | 58-93 | 2319 | 185-3089 |
| | mean | 18.4 | 7-27 | 284.8 | 73.9 | 50-93 | 2274 | 185-3151 |
